# Wolfram Syndrome Type I Case Report and Review—Focus on Early Diagnosis and Genetic Variants

**DOI:** 10.3390/medicina60071064

**Published:** 2024-06-28

**Authors:** Alexandru Daniel Jurca, Larisa Bianca Galea-Holhos, Aurora Alexandra Jurca, Diter Atasie, Codruta Diana Petchesi, Emilia Severin, Claudia Maria Jurca

**Affiliations:** 1 Department of Preclinical Disciplines, Faculty of Medicine and Pharmacy, University of Oradea, 410081 Oradea, Romania; alexjurca@uoradea.ro (A.D.J.); petchesidiana@uoradea.ro (C.D.P.); claudiajurca@uoradea.ro (C.M.J.); 2 Department of Morphological Disciplines, Faculty of Medicine and Pharmacy, University of Oradea, 410081 Oradea, Romania; 3 Faculty of Medicine and Pharmacy, University of Oradea, 410081 Oradea, Romania; jurca.auroraalexandra@student.uoradea.ro; 4 Departament II Medical Clinic, Faculty of Medicine, University “Lucian Blaga of Sibiu”, Lucian Blaga Street 2A, 550169 Sibiu, Romania; atasie.diter@ulbsibiu.ro; 5 Regional Center of Medical Genetics Bihor, County Emergency Clinical Hospital Oradea (Part of ERN-ITHACA), 410469 Oradea, Romania; 6 Genetics Department, “Carol Davila” University of Medicine and Pharmacy, 020027 Bucharest, District 2, Romania

**Keywords:** Wolfram syndrome type 1, optic atrophy, insulin-requiring diabetes mellitus, sensorineural deafness

## Abstract

*Background and Objectives*: Wolfram syndrome type 1 (OMIM# 222300; ORPHAcode 3463) is an extremely rare autosomal recessive syndrome with a 25% recurrence risk in children. It is characterized by the presence of juvenile-onset diabetes mellitus (DM), progressive optic atrophy (OA), diabetes insipidus (DI), and sensorineural deafness (D), often referred to by the acronym DIDMOAD. It is a severe neurodegenerative disease with a life expectancy of 39 years, with death occurring due to cerebral atrophy. For a positive diagnosis, the presence of diabetes mellitus and optic nerve atrophy is sufficient. The disease occurs because of pathogenic variants in the *WFS1* gene. The aim of this article is to present a case report of Wolfram Syndrome Type I, alongside a review of genetic variants, clinical manifestations, diagnosis, therapy, and long-term management. Emphasizing the importance of early diagnosis and a multidisciplinary approach, the study aims to enhance understanding and improve outcomes for patients with this complex syndrome. *Materials and Methods*: A case of a 28-year-old patient diagnosed with DM at the age of 6 and with progressive optic atrophy at 26 years old is presented. Molecular diagnosis revealed the presence of a heterozygous nonsense variant WFS1 c.1943G>A (p.Trp648*), and a heterozygous missense variant WFS1 c.1675G>C (p.Ala559Pro). *Results*: The molecular diagnosis of the patient confirmed the presence of a heterozygous nonsense variant and a heterozygous missense variant in the *WFS1* gene, correlating with the clinical presentation of Wolfram syndrome type 1. Both allelic variants found in our patient have been previously described in other patients, whilst this combination has not been described before. *Conclusions*: This case report and review underscores the critical role of early recognition and diagnosis in Wolfram syndrome, facilitated by genetic testing. By identifying pathogenic variants in the *WFS1* gene, genetic testing not only confirms diagnosis but also guides clinical management and informs genetic counseling for affected families. Timely intervention based on genetic insights can potentially reduce the progressive multisystem manifestations of the syndrome, thereby improving the quality of life and outcomes for patients.

## 1. Introduction

Wolfram syndrome (WS) is an extremely rare disease with an incidence of 1 in 160,000–770,000 [1]. In November 2023, Orphanet Report Series showed an estimated European prevalence data of 0.62 per 100,000 [2].

It was first described in 1938 by Wolfram and Wagener in four siblings who presented with juvenile-onset diabetes mellitus (DM) and optic atrophy (OA) [3]. There are three known types of Wolfram syndromes: WFS1 (OMIM #222300), WFS2 (OMIM #604928), and the mitochondrial form (OMIM #598500). The most well-known and the most common type is type 1 (WFS1), which arises from various mutations in the *WFS1* gene. Type 2 (WFS2) is much rarer, inherited in an autosomal recessive manner, and is due to pathogenic variants in the *CISD2* gene (CDGSH iron–sulfur domain-containing protein 2). The latest international classification of diseases (ICD-11) included WS in subcategory 5A16.1, categorizing it as a rare type of DM [4]. The *WFS1* gene encodes wolframin, a glycoprotein with a major role in protein folding at the endoplasmic reticulum (ER) and maintaining calcium homeostasis at the same level. Wolframin is a complex protein containing 890 amino acids and is predominantly expressed in pancreatic muscle cells, nerve cells, the eyes, the inner ear, as well as in the liver and kidneys [5]. Various mutations in the *WFS1* gene, such as mismatch, insertion, transversion, or transition, cause a significant accumulation of misfolded proteins in the ER, ultimately leading to ER stress [6].

The clinical picture includes juvenile-onset diabetes mellitus, progressive optic atrophy with gradual loss of visual acuity followed by loss of color vision, central diabetes insipidus, deafness, and progressive neurodegeneration. For a positive diagnosis, the presence of DM and optic atrophy is sufficient, as these are the most encountered manifestations [7]. While diabetes mellitus can be controlled medically, the progression of neurodegeneration cannot be halted, as there is currently no medication for this. It is a severe disease with an average survival of 39 years, primarily due to brainstem atrophy leading to severe respiratory failure [8,9]. It has a significant emotional impact on both diagnosed patients and their families. Therefore, WS requires careful clinical monitoring to alleviate suffering and improve the quality of life of these patients.

The aim of this article, through the detailed case report presented by the authors, is to emphasize the importance of the early diagnosis of WS and to highlight the importance of a multidisciplinary team in the proper management of this pathology.

## 2. Materials and Methods

### 2.1. Case Report

The authors present the case of a 28-year-old patient who came to our Regional Center of Medical Genetics in June 2022, referred by an ophthalmologist for suspected Leber hereditary optic neuropathy. The family history was negative for chronic or genetic conditions.

Past medical history: At the age of 6, the patient was diagnosed with type 1 diabetes mellitus and has been on insulin replacement therapy since. Insulin treatment involves a combination of fast-acting and long-acting insulin. The current therapeutic regimen is as follows: Insulin Aspart (Fiasp) 15 IU, three times a day before meals, subcutaneous (fast-acting), and insulin Glargine (Lantus) 15 IU once a day in the evening at 21:00, (long-acting). Since 2017, the patient has been monitored by the ophthalmology service, initially with myopia, later with glaucoma followed by papillary discoloration and optic atrophy (2022). The anamnestic evaluation revealed progressive bilateral visual loss from the age of 17. Progressively, visual acuity decreased, and at the time of presentation in our service, the diagnosis was glaucomatous optic neuropathy and optic nerve atrophy. 

Since 2022, the patient has been under the care of the psychiatry service for depression, sleep disorders, and anxiety, for which he is receiving medication.

### 2.2. Laboratory Investigations

The main lab tests performed involved carbohydrate, lipid (cholesterol, lipids, and triglycerides), protein (proteinemia, serum protein electrophoresis), mineral (calcemia, phosphatemia, and magnesemia) metabolisms, electrolytes (Na, K), and 24 h urinary excretions.

### 2.3. Imagistic Investigations

An MRI of the skull and an ocular coherence tomography (OCT) scan were performed.

### 2.4. Molecular Investigations

Total genomic DNA was extracted from the biological sample using a bead-based method. The quantity of DNA was assessed using a fluorometric method. Following this assessment, the qualified genomic DNA sample was randomly fragmented using non-contact, isothermal sonochemistry processing. Molecular tests were performed at Blueprint Genetics.

The Blueprint Genetics Whole Exome Plus Test (version 3, February 2023) involves a sequence analysis of all protein-coding genes in the proband’s genome, along with Whole Exome Deletion/Duplication (CNV) Analysis. The sequencing library was prepared by attaching sequencing adapters to both ends of the DNA fragments. The libraries were then size-selected using a bead-based method to ensure optimal template size and amplified by polymerase chain reaction (PCR). Regions of interest (exons and intronic targets) were captured using a hybridization-based target capture method. The quality of the completed sequencing library was controlled by verifying the correct template size and quantity and eliminating leftover primers and adapter–adapter dimers. Sequencing libraries that passed quality control were sequenced using Illumina’s sequencing-by-synthesis method with paired-end sequencing (2 × 150 bases).

Primary data analysis, which converts images into base calls and associated quality scores, was performed by a sequencing instrument using Illumina’s proprietary software, producing CBCL files as the final output. Bioinformatics and quality control were conducted as follows: read alignment was performed using the Burrows–Wheeler Aligner software 0.7.17. Duplicate read marking, local realignment around indels, base quality score recalibration, and variant calling were performed using GATK algorithms (Sentieon) for nDNA. Variant data were annotated using various tools (VcfAnno and VEP) and public variant databases including gnomAD, ClinVar, and HGMD.

The median sequencing depth and coverage across the target regions for the tested sample were calculated based on MQ0 aligned reads. The sequencing run included in-process reference samples for quality control, which passed thresholds for sensitivity and specificity. The patient’s sample underwent thorough quality control measures, including assessments for contamination and sample mix-up. Copy number variations (CNVs), defined as single exon or larger deletions or duplications (Del/Dups), were detected from the sequence analysis data using a proprietary bioinformatics pipeline. The difference between observed and expected sequencing depth at the targeted genomic regions was calculated, and regions were segmented based on variable DNA copy numbers. Expected sequencing depth was determined using other samples processed in the same sequence analysis as a reference. The sequence data were adjusted for variations in guanine and cytosine content.

Interpretation: The pathogenicity of identified variants was assessed based on predicted consequences, biochemical properties of the codon change, evolutionary conservation, and data from population and mutation databases such as the 1000 Genomes Project, gnomAD, ClinVar, and HGMD Professional. For missense variants, in silico prediction tools such as SIFT, PolyPhen, and MutationTaster 2 were used for classification. Additionally, the clinical relevance of any identified CNVs was evaluated through literature and databases including the 1000 Genomes Project, the Database of Genomic Variants, ExAC, gnomAD, and DECIPHER. For mtDNA variants, specific databases like Mitomap, HmtVar, and 1000 G were consulted. The clinical evaluation team assessed the pathogenicity of the identified variants by reviewing the patient’s referral information, relevant literature, and manually inspecting sequencing data if necessary. The reporting followed HGNC-approved gene nomenclature and HGVS mutation nomenclature guidelines. Likely benign and benign variants were not reported. Variants in genes with unestablished disease associations were considered potentially disease-causing using a specific evaluation scheme.

Sequence analysis using Blueprint Genetics (BpG) Whole Exome Plus identified that the patient was a compound heterozygote for WFS1, with a heterozygous nonsense variant WFS1 c.1943G>A, p.(Trp648*), and a heterozygous missense variant WFS1 c.1675G>C, p.(Ala559Pro). Both allelic variants found in our patient have been previously described in other patients, whilst this combination has not been described before.

## 3. Results

### 3.1. Clinical Evaluation of the Patient Land Aboratory Investigations

The phenotypic characteristics at the age of 28 include a normal stature (166 cm) and weight (66 kg). The patient wears glasses.

Biochemical, hematological, and hormonal (thyroid hormones) analyses revealed slightly elevated blood glucose levels of 162 mg/dL (reference value, 74–100 mg/dL) (interpreted in the context of diabetes mellitus), slightly low ionized calcium at 3.85 mg/dL (reference value, 4.2–5.20), LDL cholesterol at 199 mg/dL (reference value, 10–100 mg/dL), and normal serum electrolytes (Na, K) with a glycosylated hemoglobin level of 6.1%. The urine examination and 24 h urine output showed normal findings.

### 3.2. Opthalmological Examination

The patient was first evaluated in 2017 when he was diagnosed with myopia (the patient complained of blurry vision), corrected with glasses but without improvement in vision; progressively, the presence of glaucoma and then papillary discoloration was noted. He underwent periodic ophthalmological examinations. His pupils were isochoric, and no relative afferent pupillary defect was observed. There were no ocular motility problems in any quadrant during the examination, and no dyschromatopsia was detected using the Ishihara test.

Refraction was assessed using a Huvitz Autorefractor/Keratometer HRK-8000A. (Argus Optik, Cluj-Napoca, România) Visual acuity was measured in a 5 m long room for each eye, both without correction and with correction, using a Snellen visual chart. This was followed by cycloplegia using Cyclopentolate hydrochloride drops (SC Rompharm SRL, Otopeni, Romania), administered three times at ten-minute intervals. Cyclopentolate drops were instilled in the inferior subconjunctival sac after confirming that the patient had no history of seizures. Each drop of Cyclopentolate contained 0.3 mg of the active substance.

The Best Corrected Visual Acuity (BCVA) in 2022 for the right eye (RE) was 0.6 with difficulty and 0.7 for the left eye (LE). The refraction without cycloplegia for the RE was −0.50 sph, −0.75 cyl/130°, and for the LE was −0.25 sph, −0.75 cyl/15°. Cycloplegic refraction was measured 40 min after the instillation of the first drop of Cyclopentolate, with the following diopters: for the RE, +0.0 SPH, −0.50 cyl/129°, and for the LE, +0.25 sph, −1.0 cyl/11°. Intraocular pressure was measured with a Goldmann applanation tonometer. The RE IOP was 10 mmHg, and the LE IOP was 12 mmHg; these parameters remained almost constant at rest.

**Figure 1:**
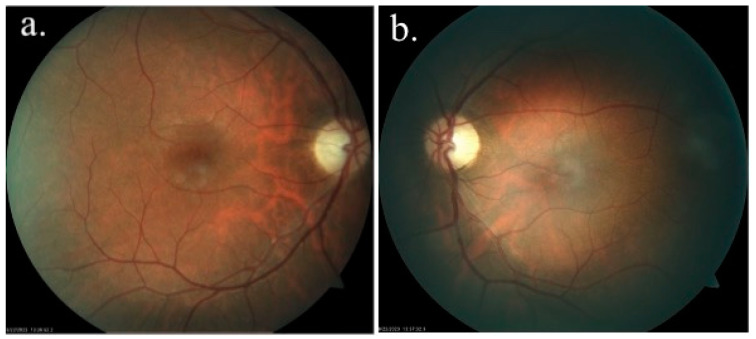
Eye fundus examination: (**a**) right eye; (**b**) left eye. Both optic nerves were pale, and the foveolar reflex was absent.

Biomicroscopy was performed only after complete cycloplegia, using a Takagi 700 GL NSW slit-lamp microscope, first in 2022, and then at regular intervals of 3–6 months. An anterior pole examination revealed clear corneas in both eyes and clear lenses in each eye. A posterior pole examination revealed a slightly pale optic nerve and the absence of the foveolar reflex in both eyes. Neither eye showed signs of diabetic retinopathy (Figure 1).

**Figure 2:**
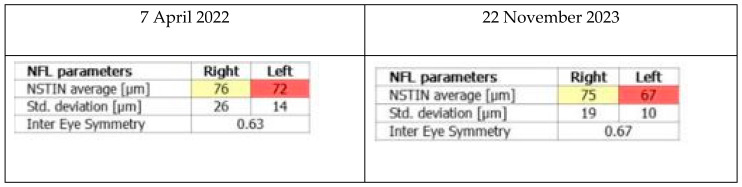
Retinal nerve fiber layer (RNFL) thickness was evaluated with optical coherence tomography (OCT). Average RNFL thickness and RNFL symmetry did not have any significant changes during the follow-up period.

A Spectral-Domain OCT examination of the optic nerves and retina was performed using an Optopol REVO 80 (Optopol Technology Sp. z o.o.ul. Zabia, Polska) (Figure 2).

**Figure 3:**
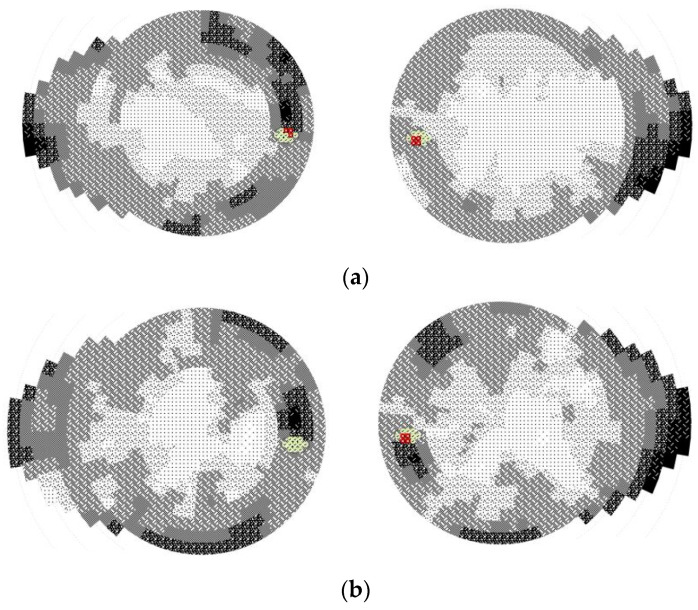
Visual field: the RE enlarged blind spot, LE nasal step (**a**) in 2022; (**b**) 2023: enlarged blind spot in both eyes.The colors red and green are often used to represent different aspects of the test results: **Red**: Red usually indicates areas where there is a significant loss of visual sensitivity. In some tests, red might highlight points where the patient’s responses were abnormal, indicating a potential defect in the visual field. **Green**: Green often represents areas where the visual sensitivity is within normal limits. Green areas indicate points where the patient’s responses were normal, suggesting no significant defects in those parts of the visual field.

The visual field test performed in 2022 on the RE and LE revealed the following: an enlarged blind spot on the RE with a nasal step and superior arcuate defect and a slightly inferior arcuate defect. The visual field test on the LE showed a nasal step. In 2023 (the RE and LE), visual field showed an enlarged blind spot on both eyes, alongside with a superior and inferior arcuate defect and nasal step. The last check-up was in 2023 November, and there were no changes in the patient’s refraction, but the BCVA OU was 0.4 (Figure 3). Although the BCVA progressively decreased, the visual field did not undergo extremely severe changes.

Other interdisciplinary assessments, including ENT evaluation combined with audiometry and psychiatric evaluation, conducted periodically, have not revealed any pathological changes at present.

### 3.3. Imagistic Investigations

#### 3.3.1. Abdominopelvic Ultrasound

The abdominopelvic ultrasound was within normal limits.

#### 3.3.2. MRI Skull

There was a non-specific gliotic lesion measuring 5 mm in the left frontal posterior white matter and another measuring 4 mm in the right parietal lobe. The MRI angiography showed no signs of aneurysmal stenosis or dilatation in the cerebral arteries of the Willis polygon and the vertebrobasilar system.

### 3.4. Molecular Investigations

A sequence analysis using Blueprint Genetics (BpG) Whole Exome Family Plus identified a heterozygous nonsense variant WFS1 c.1943G>A, p.(Trp648*) and a heterozygous missense variant WFS1 c.1675G>C, p.(Ala559Pro). 

The mother is heterozygous for WFS1 c.1943G>A, p.(Trp648*), and the father is heterozygous for the WFS1 c.1675G>C, p.(Ala559Pro). His brother and his wife both were negative for the *WFS1* gene.

## 4. Discussion

### 4.1. Genetics

#### 4.1.1. *WS1* Gene and Wolframin

The *WFS1* gene was identified in 1998 and is located on the short arm of chromosome 4 (4p16.1), containing eight exons. Although genomic variants can span the entire length of the gene, pathogenic and likely pathogenic variants are concentrated in exon 8 (88%) of patients [10]. Extremely few mutational variants are found in other exons of the gene [11]. It encodes a transmembrane protein, wolframin. Pathogenic variants of the *WFS1* gene are most commonly associated with the autosomal recessive form of Wolfram syndrome (WFS1), autosomal dominant-Wolfram-like syndrome (WFSL, MIM #614296), and WFS1-related low-frequency sensorineural hearing loss (LFSNHL, DFNA6/14/38, and MIM #600965). Sometimes it is also associated with congenital nuclear cataract-41, with recent studies showing that certain pathogenic variants of the *WFS1* gene are associated with non-syndromic optic atrophy [7,12].

Mutations in the *WFS1* gene typically have a significant impact on the clinical phenotype, and homozygosity or compound heterozygosity are absolutely mandatory for diagnosis. Mutations can have multiple effects at the RNA level. RNA expression is much lower in the case of missense mutations, thus demonstrating a much more complex pathogenic mechanism [13].

These mutational variants accentuate protein instability by halving its normal lifespan compared to the wild-type protein [14]. To date, over 464 mutations of WFS1 have been reported in the Human Gene Mutation Database, making it difficult to establish a genotype–phenotype correlation [15].

Wolframin is a transmembrane protein (with nine domains) consisting of 890 amino acids. It is located in the endoplasmic reticulum membrane where it plays a role as a protein chaperone and/or in membrane trafficking. It intervenes in the mechanism of protein folding and post-translational secretion, calcium homeostasis at the endoplasmic reticulum level, as well as in insulin biosynthesis and secretion. However, the exact mechanism of its action is not fully understood [16,17,18]. It has a complex structure organized in tetramers and has a molecular weight of 400 kD. It contains a C-terminal end, which is hydrophilic, positioned in the endoplasmic reticulum lumen, and an N-terminal end in the cytoplasm. Its distribution in the body is ubiquitous, but with higher concentrations in the pancreas in beta cells, brain, eyes, and heart [19,20,21].

The malfunction of wolframin caused by mutations in the *WFS1* gene results in abnormal protein folding in the ER followed by excessive accumulation at this level, leading to ER stress. As the number of misfolded proteins increases, a network of signaling pathways called the unfolded protein response (UPR) is activated and stimulated, attempting to restore ER homeostasis by increasing the expression of molecular chaperones, reducing protein translation, and degrading those misfolded proteins accumulated abnormally in the ER [22]. In situations where the endoplasmic reticulum is constantly and progressively subjected to stress, either as a result of physiological processes such as postprandial insulin synthesis or as a result of pathological processes such as gene mutations, cancer, inflammatory or infectious diseases, the UPR stimulates and ultimately induces cell death (cell apoptosis) [21,23,24,25,26]. The C-terminal end of the protein is the major site where most missense mutations occur, affecting the translation of the last 10–15 amino acids, thus leading to the clinical presentation of the disease, confirming the major functional role of the C-terminal end [27,28]. Mutations can also occur at the N-terminal end (cytoplasm) or at the membrane level [29,30].

Although wolframin is ubiquitously expressed, on the one hand, there are certain tissues where it is expressed at much higher levels, such as in pancreatic beta cells and the brain, and on the other hand, there are tissues where it has a lower level of expression (e.g., kidney and blood) [19]. In the pancreas, wolframin contributes to the folding of proinsulin (the precursor of insulin) into the mature hormone, insulin; in the inner ear, it maintains calcium and other ions at optimal levels essential for hearing. Since WFS1 is expressed in the brain, it is closely associated with neuropsychiatric disorders. Initial studies have shown that carriers of WFS1 heterozygosity have an increased risk of mood disorders, a finding later confirmed by additional studies [31,32,33]. Furthermore, some studies have shown that certain mutational variants of WFS1 are correlated with a higher rate of suicide [34,35], with the explanation being given by the ER stress system, which seems to be the central key determining the deficiency in emotion regulation.

Moreover, this variability in the manifestation of neuropsychiatric issues among these individuals and the heightened susceptibility to emotional disorders in WFS1 carriers, as previously mentioned [13], can be elucidated by the dual involvement of WFS1 in ER stress and cell survival, thereby positioning this gene as a pivotal mediator between the brain and the endocrine pancreas.

#### 4.1.2. Variants: c.1943G>A, p.(Trp648*) and c.1675G>C, p.(Ala559Pro)

##### c.1943G>A, p.(Trp648*) Variant

This mutational variant has been described in four individuals heterozygous for it (gnomAD). A premature stop codon occurs in the last exon of the *WFS1* gene, causing the transcript to escape nonsense-mediated decay; this truncation leads to the loss of 243 C-terminal amino acids. In the literature, this variant has been reported in two patients in a compound heterozygous state; it was reported together with a frameshift variant in two patients with Wolfram syndrome. Another variant leading to a premature stop codon at the same position, WFS1 c.1944G>A, p.(Trp648*), has been reported as compound heterozygous (confirmed in trans) with WFS1 p.(Gly695Val) in three affected siblings with Wolfram syndrome. This variant has been entered into ClinVar by additional clinical testing laboratories under the variation ID 1697349. 

Table 1 details these associations. 

##### c.1675G>C, p.(Ala559Pro)

This variant is not present in gnomAD and has been reported in compound heterozygosity (unconfirmed as being in trans) with a truncated variant in a patient with type 1 diabetes and optic atrophy [36]. In the same codon, another missense variant p.(Ala559Asp) has been reported in an individual with phenotypes related to WFS1, highlighting the functional importance of the affected amino acid [37]. The patient presented by the authors is compound heterozygous for the two variants: c.1943G>A, p.(Trp648*) and c.1675G>C, p.(Ala559Pro). This combination has not been described in the current literature. The nonsense variant causing a premature stop codon was inherited from the mother, while the other missense variant was inherited from the father.

### 4.2. Clinical Aspects

#### 4.2.1. General View

Wolfram syndrome presents a complex symptomatology and can be described as a spectrum disorder. For a positive diagnosis, it is sufficient for the patient to exhibit diabetes mellitus diagnosed in early childhood, around the age of 6, and bilateral optic atrophy (OA) with progressive evolution, which typically occurs in most cases around the age of 15 [2,38]. In addition to these aspects, patients also present with diabetes insipidus, sensorineural deafness, urinary tract anomalies, and neurodegeneration, which usually manifest later, in adulthood [7,39].

As the clinical picture includes a constellation of signs and symptoms, Rigoli et al. suggested that for a positive clinical diagnosis, two major criteria (DM and OA) or one major criterion with two minor ones are required (Table 2) [1,39]. De Hereira et al., in a comprehensive study involving 412 reported WS1 patients, found that the most common manifestations are DM (98.21%) followed by OA (82.1%). The average age of death is around 30 years, with respiratory failure caused by respiratory center atrophy being the most frequent cause [7].

The primary differential diagnosis often involves mitochondrial diseases, particularly Leber optic atrophy, alongside other conditions like the autosomal dominant form of optic atrophy, Friedreich ataxia, Bardet–Biedl syndrome, and Alström syndrome [1]. Our patient meets the two major criteria for WS: insulin-dependent diabetes mellitus (DM) and progressive optic atrophy, which sufficiently support the diagnosis.

#### 4.2.2. Endocrine Impairment in WS

##### Juvenile DM

This pathology is the most encountered in WS, typically presenting around the age of 6 (ranging from 3 to 16 years) [40]. This form of diabetes is non-autoimmune, insulin-dependent, and not associated with the HLA system, which can initially lead to misdiagnosis as type 1 diabetes mellitus [41,42].

The pathophysiological mechanism of DM in Wolfram syndrome involves the expression of the *WFS1* gene in pancreatic beta cells. If a mutation occurs in WFS1, wolframin becomes non-functional, resulting in the chronic accumulation of misfolded proteins in the ER. Additionally, WFS1 is upregulated during insulin secretion, influencing the folding and processing of proinsulin prohormone in the ER of pancreatic β cells [24,43]. The endoplasmic reticulum is where insulin synthesis and proinsulin folding take place, and the misfolding of proteins in the ER leads to persistent stress, resulting in chronic inflammation and ultimately cellular apoptosis [38,44,45,46]. All these changes, including malfunction of beta pancreatic cells accompanied by cellular apoptosis, lead to a reduction in the number of pancreatic β cells, ultimately resulting in decreased insulin production and hyperglycemia, leading to diabetes [47]. In a vicious cycle, constant and progressive hyperglycemia exacerbates the malfunction of pancreatic β cells by intensifying ER stress, ultimately leading to insulin gene suppression [48].

Clinical signs that differentiate DM in WS from type 1 DM include an early onset, a very rare presence of autoantibodies, extremely rare episodes of ketoacidosis, lower daily insulin doses, and much rarer hypoglycemic episodes [7,23]. Microvascular complications are also much less common compared to type 1 DM. Glycemic control is also much better in these patients, with lower glycated hemoglobin values. Cano et al., in a study involving 26 patients treated with insulin for diabetes associated with WS and 56 patients diagnosed with type 1 DM, showed that HbA1C values were much lower in the WS patient group (7.72 ± 0.21%) compared to HbA1C 8.99 ± 0.25% in patients with type 1 DM; insulin doses were also much lower in WS patients (0.71 ± 0.07 IU/kg/day) compared to 0.88 ± 0.04 IU/kg/day in patients with type 1 DM [49]. Endocrinologists and other specialists should consider these differences between the two forms of diabetes to avoid a misdiagnosis of WS, which could have severe consequences on the patient’s health [39]. Our patient was diagnosed and treated for type 1 DM from the age of 6. He is currently undergoing treatment with the rapid-acting insulin Aspart (Fiasp), 15 IU/3×/day subcutaneously, combined with Glargine (Lantus) 15 IU/1×/day in the evening, falling within the insulin requirements reported in previous studies for the same category of patients. The glycemic control is also good, with an HbA1C of 6.1% (the target value is under 7%).

##### Diabetes Insipidus (Arginine Vasopressin Deficiency Syndrome)

Diabetes insipidus (arginine vasopressin deficiency syndrome) is considered a major diagnostic criterion. It frequently occurs in Wolfram syndrome, with an average onset age of 14 years (ranging from 3 months to 40 years). Diagnosis at onset can be challenging due to partially present symptoms. Therefore, it is crucial to ensure proper monitoring of all Wolfram syndrome patients to promptly establish this diagnosis [7].

##### Hypogonadism

In some patients, hypogonadism may occur, resulting in fertility problems. Men affected by this condition may encounter erectile dysfunction, whereas women may experience disruptions in their menstrual cycle [49].

#### 4.2.3. Ocular Involvement: Optic Atrophy

Optic atrophy is an essential criterion for WS diagnosis. The onset of ocular involvement typically occurs around the age of 11 (ranging from 6 to 19 years) [de Hereira]. Ocular symptoms include cataracts, abnormal pupil reflexes, glaucoma leading to progressive visual acuity loss and subsequent color vision impairment, retinal pigment changes, and progressive glaucoma [1,38,39]. Patients require frequent monitoring, with high-definition optical coherence tomography (OCT) and/or magnetic resonance imaging (MRI) being the preferred investigations to observe changes in retinal thickness. In these patients, retinal thickness is not as pronounced as in type 1 DM patients [50,51]. Our patient was initially diagnosed with glaucoma at the age of 26, followed by papillary discoloration (optic atrophy). The current treatment focuses on glaucoma management with Brimonidine one drop twice daily for 3 months and Brinzolamide and timolol (Azarga) one drop twice daily. Additionally, the treatment to improve visual acuity includes Citicoline (Neukron) once daily and Idebenone as antioxidants.

#### 4.2.4. Hearing Impairment

More than 50% of WS patients have associated deafness, with the average onset age being 12.5 years (a range of 5–39 years) [52,53]. In most cases, it is asymptomatic and is detected through audiometric tests [54].

#### 4.2.5. Neurological and Psychiatric Abnormalities

##### Neurological Problems

Neurological problems are consistently observed in individuals with WS. While some studies report their prevalence in up to 50% of patients, others suggest it could be as high as 70%. The most common neurological manifestation is cerebellar ataxia, although occurrences of epilepsy, areflexia, myoclonus, and respiratory center atrophy are less frequent. These neurological disorders significantly contribute to morbidity and mortality, as areflexia increases the risk of aspiration pneumonia, and respiratory center atrophy heightens the risk of central apnea [7,9,55]. Additionally, patients may experience autonomic symptoms such as sweating disorders, disrupted thermoregulation, orthostatic hypotension, alternating periods of constipation and fecal incontinence, as well as headaches [7,56].

##### Psychiatric Abnormalities

Genetic factors play a significant role in the development of psychiatric disorders, with increased susceptibility often linked to specific genes. In Wolfram syndrome, psychiatric symptoms typically emerge in young adulthood, affecting over 50% of patients by the age of around 20.5 years [7]. These symptoms commonly include depression, anxiety, panic attacks, and sleep disturbances, sometimes escalating to severe depressive episodes with a risk of suicide [57,58].

Research has indicated that heterozygosity of the *WFS1* gene raises the risk of depression, as demonstrated by Swift et al. in a study involving 25 WS patients, where 11 relatives exhibited psychiatric disorders. Unlike in type 1 diabetes mellitus, where psychiatric issues, particularly depression, often stem from insulin treatment, psychiatric symptoms in WS are more prevalent (30%) and associated with ER stress [59]. The patient described by the authors has been under psychiatric care since 2022 for severe sleep disturbances, anxiety, and depression. Initially treated with Lorazepam (Anxiar), Clonazepam (Rivotril), and Genodorm, their treatment was adjusted due to persistent symptoms. Currently, they are receiving antidepressant therapy with Duloxetine (Cymbalta) and Quetiapine ½ tablet, resulting in improved anxiety levels and sleep patterns (now sleeping uninterrupted for 5 h per night).

### 4.3. Genotype–Phenotype Correlation

Given the large number of pathogenic variants described in WFS1—over 490 in international databases [60]—it is difficult to clearly establish a genotype–phenotype correlation. Although some studies have attempted to highlight such correlations, the most relevant is de Heredia’s study, which included 412 patients with WS1 and identified 178 mutations in the *WFS1* gene [7]. The difficulty in establishing a genotype–phenotype correlation arises from the complexity of the WFS1 molecular structure, the highly variable clinical presentations even among individuals within the same family, and the small patient cohorts used in the studies. At the same time, the type and location of the pathogenic variant do not predict the phenotype [17,61]. It appears that compound heterozygotes are at a higher risk for DM, deafness, and psychiatric disorders [7,62]. Cano et al., in a study including 12 patients from 11 families with WS, and Rigoli et al., in a study including 45 WS patients, demonstrated that patients homozygous or compound heterozygous for two inactivating mutations had an early onset of DM and OA [17,49].

### 4.4. Treatment and Follow-Up

At present, there is no curative treatment to halt the neurodegenerative changes in WS; only supportive care is available. For instance, insulin therapy for correcting blood glucose levels remains the primary option for children and adolescents. Clinical studies, some in more advanced phases and others still in the preclinical stage, are primarily focused on restoring ER calcium homeostasis, optimizing the structure of wolframin using various chaperones, ER stress modulation, regenerative peptide drugs, and gene therapy. These represent the main therapeutic strategies [22].

#### 4.4.1. Stabilizers of Calcium Homeostasis

In WS, elevated levels of cytoplasmic calcium led to the activation of calpain, a cytosolic cysteine protease involved in cell growth, differentiation, and death. Its activity is closely linked to cytoplasmic calcium levels. When calpain is hyperactivated, cell death occurs. Treatment with calpain inhibitors is one of the therapeutic targets under investigation. Dantrolene is a calpain inhibitor that suppresses calcium transport from the ER to the cytoplasm (thus reducing cytoplasmic calcium levels) and simultaneously inhibits ryanodine receptors in the ER. It is being investigated in a phase Ib/IIa study [ClinicalTrials.gov identifier: NCT02829268; accessed on 10 May 2024]. Dantrolene is FDA-approved as a medication for malignant hyperthermia and muscle spasms. Its role in regulating, or more precisely, modulating calcium levels is crucial in the disease progression of WS patients and could potentially become an effective treatment method in the future. The protective effect of dantrolene treatment on cells with WFS1 deficiency suggests that dysregulated cellular calcium homeostasis plays a role in disease progression [63].

#### 4.4.2. Chaperone for Optimizing the Structure of Wolframin

Chaperones play a role in stabilizing the protein structure during its folding process, thereby facilitating the movement of mutant proteins at the ER level. Currently, the FDA has approved two chaperones, 4-phenylbutyric acid (PBA) and tauroursodeoxycholic acid (TUDCA), which can improve the function of pancreatic β cells and prevent ER stress-mediated pancreatic cell apoptosis [1].

#### 4.4.3. ER Stress Modulators and Regenerative Peptide Drugs

By suppressing or halting ER stress, certain pathways that lead to cell survival are activated. Currently, the role of sodium valproate is being studied, as it increases the level of p21 (a cyclin-dependent kinase inhibitor), providing protective effects against cellular stress and exhibiting an anti-apoptotic role [64,65]. Sodium valproate appears to modulate stress in neuronal cells by inducing the expression of WFS1 through the regulation of an ER chaperone protein called glucose-regulated protein 94 (GRP94). Currently, there is an ongoing evaluation in a phase II study, double-blind, placebo-controlled trial: “Efficacy and Safety Trial of Sodium Valproate, in Paediatric and Adult Patients with Wolfram Syndrome” [66].

#### 4.4.4. Glucagon-like Peptide-1 Receptor (GLP-1R) agonists

GLP-1 is a peptide secreted by intestinal cells that intervenes in numerous physiological processes. Its major role is in lowering blood sugar levels, but it also contributes to reducing the apoptosis of pancreatic β cells mediated by ER stress while simultaneously exerting neuroprotective effects both centrally and peripherally [67].

Glucagon-like peptide-1 receptor agonists (GLP-1RA), commonly used for type II DM treatment, have a positive effect on both diabetes and neurodegeneration occurring in WS. They stimulate insulin secretion in pancreatic beta cells and inhibit glucagon secretion from pancreatic alpha cells, reduce appetite, and delay stomach emptying. Through all these actions of GLP-1RA, ER stress is reduced in WS patients [68,69,70].

In 2006, Yusta et al. showed that treatment with exenatide (an incretin mimetic agent) significantly reduced insulin requirement by up to 70% in patients with type 2 DM, and in 2018, Kondo et al. also showed that patients with type 2 DM treated with liraglutide, a long-acting agonist of GLP-1R, had better glycemic control, with insulin dose requirements decreasing by up to 20% in WS [71,72,73]. In the pediatric population, Frotino et al. demonstrated the effectiveness of liraglutide in an off-label study that included four patients aged 10–14 years over a period of 8–27 months. The effects were positive, insulin doses decreased, no changes in pancreatic enzymes occurred, the area under the curve of C-peptide varied between 81% and 171% of the initial value, and in terms of ocular and neurophysiological impairments, they remained constant during the follow-up period [36]. The study’s limitations were the small number of patients, short follow-up duration, and lack of genotype–phenotype correlation. The conclusion of these studies was that this treatment with GLP1-1RA, especially liraglutide, reduces the oxidative stress associated with diabetes [74,75].

#### 4.4.5. Gene Therapy

##### The Use of Adeno-Associated Viral Systems (AAVs)

The adeno-associated viral system (AAV) is utilized to transfer the wild-type *WFS1* gene into retinal ganglion cells. Given the eye’s layered anatomical structure, which allows direct access, it serves as an ideal organ for gene therapy [76]. Presently, Voretigene gene therapy stands approved for treating Leber’s hereditary optic neuropathy, marking the first approved product for this condition [77].

##### Gene Editing

As for gene editing, CRISPR technology is employed to target and correct the *WFS1* gene mutation with wild-type alleles in Wolfram syndrome.

##### Regenerative Medicine

Therapies in regenerative medicine focus on utilizing patients’ Induced Pluripotent Stem Cells (iPSCs) as another therapeutic approach. Apart from iPSCs, certain regenerative factors like mesencephalic-astrocyte-derived neurotrophic factor (MANF) are also utilized; it seems to offer protection against cellular apoptosis induced by endoplasmic reticulum stress [78,79]. However, more studies are required to establish their safety and effectiveness.

### 4.5. Follow-Up and Genetic Counselling

For the proper monitoring and evaluation of patients with Wolfram syndrome, a multidisciplinary team is extremely important. It is mandatory to conduct annual assessments for diabetes mellitus, ophthalmology, ENT (ear, nose, and throat), and nephrology. Neurological and psychiatric evaluations should also be conducted periodically, as depression and other psychiatric issues are significant components of the syndrome.

Since Wolfram syndrome is inherited in an autosomal recessive manner, the risk of having affected children is 25% if both parents are heterozygous carriers. In the specific case described by this study, as the wife does not carry an abnormal WFS1 allele, the recurrence risk for offspring being carriers is 100%.

## 5. Conclusions

Wolfram Syndrome is a rare and highly complex condition affecting multiple organs and tissues. Diagnosis can be facilitated by awareness of its major clinical criteria, despite its rarity.

Genetics significantly influences this disease, primarily through pathogenic variants in the *WFS1* gene, which lead to dysfunctional wolframin proteins and progressive symptoms. Genetic testing aids in identifying carriers.

Delayed diagnosis often results in complications and high mortality rates due to the disease’s severe progression. Therefore, early diagnosis and proper management are critical. Given the challenging prognosis, specialists must closely monitor the psychological well-being of patients.

Patients presenting with both diabetes mellitus (DM) and optic atrophy should be promptly evaluated for Wolfram syndrome. Once diagnosed, lifelong monitoring is essential. The disease profoundly impacts patients and their families, confronting them with its devastating prognosis. In a multidisciplinary approach, geneticists play an important role in diagnosis and provide genetic counseling on etiology, progression, complications, treatment options, and family planning for these patients.

## Figures and Tables

**Table 1:** c.1943G>A, p.(Trp648*) variant.

	Compound Heterozygous	
WFS1 c.1943G>A, p.(Trp648*)	WFS1 p.(Val779Gly)	PMID: 21564155
	WFS1 p.(Ser168del)	
	WFS1 p.(Gly695Val)	PMID: 9771706
	frameshift variant WFS1 p.(Met623Trpfs*2)	PMID: 30957632

**Table 2:** Major and minor diagnostic criteria for WS [39].

Major Clinical Features	Average Age of Diagnosis
Diabetes insipidus	3 months–40 years
Diabetes mellitus	3 weeks–16 years
Optic atrophy	6 weeks–19 years
Sensorineural hearing loss	5–39 years
Neurological and psychiatric problems	5–44 years
Urinary tract symptoms (neurogenic bladder, bladder incontinence, urinary tract infections)	13–33 years

## Data Availability

The data described in this study are available upon request from the corresponding author.
